# Breast cancer stage and molecular subtype distribution: real-world insights from a regional oncological center in Hungary

**DOI:** 10.1007/s12672-024-01096-9

**Published:** 2024-06-22

**Authors:** Judit Tittmann, Tamás Ágh, Dalma Erdősi, Bettina Csanády, Erika Kövér, Antal Zemplényi, Sándor Kovács, Zoltán Vokó

**Affiliations:** 1 Center for Health Technology Assessment, Semmelweis University, Üllői Str 25, Budapest, 1091 Hungary; 2 Center for Health Technology Assessment and Pharmacoeconomic Research, University of Pécs, Pécs, Hungary; 3 Syreon Research Institute, Budapest, Hungary; 4 Department of Oncotherapy, Medical School and Clinical Center, University of Pécs, Pécs, Hungary

**Keywords:** Breast cancer distribution, TNM stage, Molecular subtype, Age cohorts, Population data

## Abstract

**Objective:**

Examining the distribution of breast cancer (BC) stage and molecular subtype among women aged below (< 45 years), within (45–65 years), and above (> 65 years) the recommended screening age range helps to understand the screening program's characteristics and contributes to enhancing the effectiveness of BC screening programs.

**Methods:**

In this retrospective study, female patients with newly diagnosed BC from 2010 to 2020 were identified. The distribution of cases in terms of TNM stages, severity classes, and subtypes was analysed according to age groups.

**Results:**

A total of 3282 women diagnosed with BC were included in the analysis. Among these cases 51.4% were detected outside the screening age group, and these were characterized by a higher TNM stage compared to those diagnosed within the screening age band. We observed significantly higher relative frequency of advanced BC in the older age group compared to both the screening age population and women younger than 45 years (14.9% vs. 8.7% and 7.7%, P < 0.001). HR−/HER2− and HER+ tumours were relatively more frequent among women under age 45 years (HR−/HER2−: 23.6%, HER2+: 20.5%) compared to those within the screening age range (HR−/HER2−: 13.4%, HER2+: 13.9%) and the older age group (HR−/HER2−: 10.4%, HER2+: 11.5%).

**Conclusions:**

The findings of our study shed light on potential areas for the improvement of BC screening programs (e.g., extending screening age group, adjusting screening frequency based on molecular subtype risk status) in Hungary and internationally, as well.

**Supplementary Information:**

The online version contains supplementary material available at 10.1007/s12672-024-01096-9.

## Introduction

Breast cancer (BC) is a major public health concern with a significant impact on individuals and society. It ranks among the most frequently diagnosed cancers in women worldwide, constituting nearly one-quarter of all female cancer cases [1]. In 2020, approximately 2.3 million new BC cases and 685,000 BC caused deaths were reported worldwide [1]. Screening represents a measure of secondary prevention, capable to reduce this burden by detecting BC in asymptomatic women at an early stage, when treatment is more likely to be successful [2].

Currently, X-ray mammography serves as the standard method for BC screening [3]. However, screening guidelines vary among countries, particularly regarding the age range for screening. In Hungary, since 2002, there has been an organized, population-based breast cancer screening program, that invites women aged 45–65 years to undergo mammography biannually [4]. Despite the program’s existence for over two decades, there is still room for improvement. In 2018, Hungary’s standardized mortality rate (European standard population) for female BC exceeded the European Union (EU) average (39.0 deaths per 100,000 individuals in Hungary vs. 32.9 per 100,000 in the EU) [5]. Improving the national BC screening program could serve as a precious tool for saving lives.

Numerous studies have demonstrated that the risk of BC increases with advancing age [6–8]. The SEER (Surveillance, Epidemiology, and End Results) study has provided valuable insights into the relationship between age and BC survival. Notably, the age of 60 and above was identified as a significant and independent predictor of a poor prognosis [9]. Furthermore, women with BC, who surpass the age at which screening is offered may face a decreased survival probability and an increased likelihood of being diagnosed with advanced-stage BC [10]. Conversely, younger women under the age 40 encounter unique challenges and considerations when diagnosed with BC [11–13]. A study conducted in Hungary, revealed that there was a 6% decline in BC incidence among individuals aged 50 years and above between 2011 and 2019. However, it indicated a rise under 50 years. Specifically, there was a significant 30% increase in BC incidence among those aged 30 to 39 years [14]. Furthermore, Johansson et al. observed that mortality rate among BC patients under 40 years is higher compared to those within the screening age range [11]. Correspondingly, Kim et al. found that women under the age of 40 are more prone to aggressive forms of BC and exhibit higher BC specific mortality rates than older women [13].

Moreover, molecular subtyping has emerged as a crucial tool for BC management. Through immunohistochemistry (IHC), it is possible to differentiate four main BC subtypes based on the expression of hormone receptors (HR) (such as estrogen receptors [ER] and progesterone receptors [PR]) and the human epidermal growth factor receptor 2 (HER2). Each subgroup exhibits unique characteristics, clinical presentations, and disease progression patterns. Recent research indicates that the distribution of BC molecular subtypes may vary according to age [15–17]. Cai et al. conducted a study revealing that HR+/HER2− tumours are most prevalent among women over 60 years, while women under 40 years exhibit the lowest frequency of this subtype [15]. Conversely, the proportion of HR−/HER2− subtype among BC patients is higher in younger than in older counterparts [18]. Response to treatments and prognosis of BC are associated with the molecular subtype, therefore it is crucial to consider both age and molecular subtype in BC screening and prevention efforts.

Our objective was to gain a deeper understanding of the characteristics of the Hungarian national BC screening program by examining the distribution of BC stage and molecular subtype among women aged below, within, and above the recommended screening age range. Through this research, our ultimate goal is to improve patient outcomes and mitigate the impact of this devastating disease by identifying potential areas which may serve as targets for enhancing the BC screening program in Hungary.

## Materials and methods

### Settings and study population

For this retrospective observational study, we utilised the research database developed from the electronic medical records of the Clinical Center of the University of Pécs, which serves as one of the four regional centers for oncology patient care in Hungary. This data platform connects and stores real-word data generated during routine cancer care at the Clinical Center. Related to BC, the database includes inpatient healthcare records since 1997 and outpatient data since 2007. For our analysis, clinicopathologic data were retrieved including patients’ age at diagnosis; year of the BC diagnosis; TNM stage; ER, PR, and HER2 statuses. This study was performed in line with the principles of the Declaration of Helsinki. The utilization of this oncological database for medical and health-economic research and analytical purposes has obtained approval from the Hungarian Scientific and Research Ethics Committee (ETT TUKEB IV/4068-1/2022/EKU).

The analysis included female patients who were newly diagnosed with primary BC (identified by the International Classification of Diseases code, ICD code: C50 and D05) regardless of the indication of the mammography (i.e., screening or diagnostic) at the Clinical Center of the University of Pécs from 1st of January 2010 to 31st of December 2020. The year of the pathological diagnosis was considered as the year of diagnosis. Patients with prior history of BC or secondary BC were excluded from the study. Adhering to the protocol of the Hungarian BC screening program, the study population was divided into three age cohorts on the age of BC diagnosis: (i) women aged < 45 years (individuals younger than the recommended screening age range), (ii) women aged 45–65 years (individuals within the recommended screening age range), and (iii) women aged > 65 years (individuals older than the recommended screening age range).

### Classification of breast cancer

Classification of tumours in terms of anatomic TNM stage and molecular subtype was based on the result of histological analysis conducted at the time of BC diagnosis. Anatomic TNM stage was categorised according to the AJCC Cancer Staging Manual, 8th Edition [19]. Early-stage BC was defined as TNM stages from 0 to IIB, advanced-stage as TNM stages from IIIA to IV.

Molecular subtype of tumours was defined based on the status of HR and HER2. Tumours were classified HR positive if either ER or PR status was positive. Conversely, tumours were categorised as negative if both ER and PR status were negative. HER2 positivity was determined if immunohistochemistry (IHC) yielded 3+ results, or if IHC was 2+ and the in situ hybridization (FISH amplification) test was positive. Otherwise, HER2 was considered negative. Based on the combination of HR and HER2 status, tumours were classified into four subtypes: HR-positive/HER2-negative (HR+/HER2−), HR-positive/HER2-positive (HR+/HER2+), HR-negative/HER2-positive (HR−/HER2+), and HR-negative/HER2-negative (HR−/HER2−) subtypes.

### Statistical methods

No imputation was conducted for missing data in this analysis. First, descriptive statistics on the general characteristics (i.e., number of subjects, age at diagnosis, TNM stage, stage of BC and BC molecular subtype) of the study population were computed by age groups (i.e., patients aged < 45 years, 45–65 years and > 65 years). The number of newly diagnosed BC patients was then calculated for each age cohort and year within the study period. Next, the distributions of tumours by TNM classification, BC severity and BC molecular subtypes for each age cohort were described by aggregating data across all years studied. Subsequently, we examined whether the proportions of BC severity categories (i.e., early-stage and advanced-stage BC) and molecular subtypes (i.e., HR+/HER2−, HR+/HER2+, HR−/HER2+ and HR−/HER2−) varied across the years in the period of 2010 to 2020. We used Pearson's Chi-squared test for the analysis, first for the whole study population and then for each age cohort separately. Finally, we tested with the same Pearson's chi-squared test whether the distribution of BC severity and molecular subtype differed by age groups using all incidence cases diagnosed in the whole study period. A P-value less than 0.05 was considered statistically significant. The data analysis was performed using STATA software (version 16.1) and as a quality assurance measure, the analysis was repeated using R software (version 4.1.2).

## Results

### Patient characteristics

**Fig. 1:**
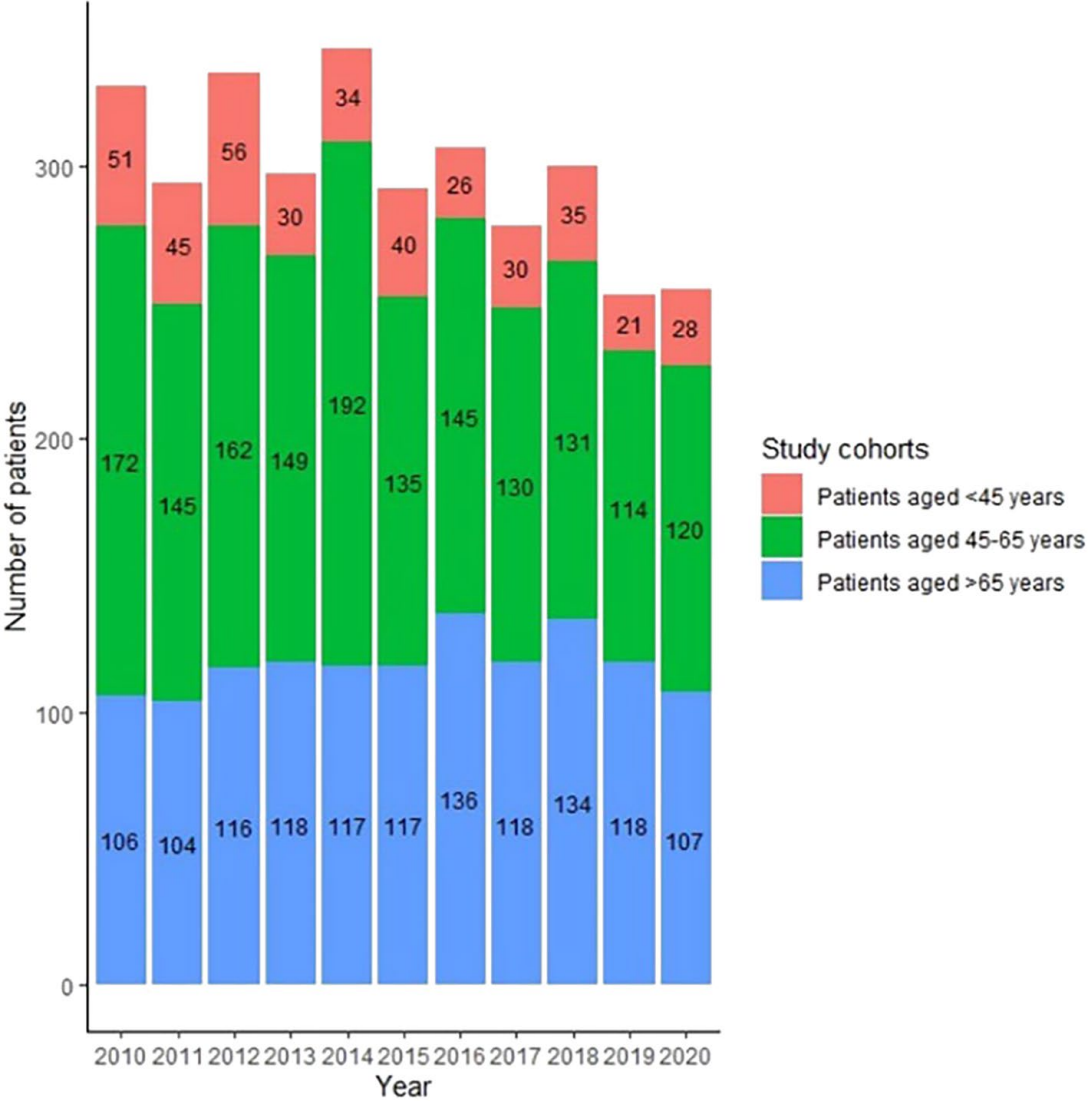
Annual number of newly diagnosed breast cancer cases during the study period

Between 2010 and 2020, a total of 3282 women were newly diagnosed with BC at the Clinical Center of University of Pécs. The average (standard deviation) number of newly diagnosed cases per year was 298 (29), with a range of 253 (in 2019) to 343 (in 2014) patients per year (Fig. 1). The general characteristics of the study sample are presented in Table 1. Women below the age of 45 years accounted for 12.1% of the study population, women aged 45–65 years, who are the target of the organised BC screening program in Hungary, represented 48.6%, and women above the age of 65 years constituted 39.3% of the included patients. The average (standard deviation) age of the study population was 61.3 (12.9) years.

**Table 1:** General characteristics of the study population

	Patients aged < 45 years	Patients aged 45–65 years	Patients aged > 65 years	Total
Study cohorts (N)	396	1595	1291	3282
Age at diagnosis (mean (SD))	38.63 (4.4)	56.6 (5.9)	73.7 (5.8)	61.2 (12.9)
TNM stage (N (%))				
0	8 (3.8)	20 (1.8)	11 (1.1)	39 (1.7)
IA	87 (41.8)	55 (50.6)	373 (37.3)	1015 (44.1)
IB	3 (1.4)	22 (2.0)	13 (1.3)	38 (1.65)
IIA	71 (34.1)	284 (25.9)	305 (30.5)	660 (28.7)
IIB	23 (11.1)	120 (10.9)	148 (14.8)	291 (12.6)
IIIA	8 (3.8)	60 (5.5)	76 (7.6)	144 (6.3)
IIIB	1 (0.5)	7 (0.6)	44 (4.4)	52 (2.3)
IIIC	6 (2.9)	22 (2.0)	28 (2.8)	56 (2.4)
IV	1 (0.5)	6 (0.5)	1 (0.1)	8 (0.3)
Missing data	188 (47.7)	499 (31.3)	292 (22.6)	979 (29.8)
Stage of BC (N (%))				
Early-stage	192 (92.3)	1001 (91.3)	850 (85.1)	2043 (88.7)
Advanced-stage	16 (7.7)	95 (8.7)	149 (14.9)	260 (11.3)
Missing data	188 (47.7)	499 (31.3)	292 (22.6)	979 (29.8)
Molecular subtype (N (%))				
HR+/HER2−	163 (55.8)	961 (72.7)	853 (78.0)	1977 (73.0)
HR+/HER2+	34 (11.6)	107 (8.1)	71 (6.5)	212 (7.8)
HR−/HER2+	26 (8.9)	77 (5.8)	55 (5.0)	158 (5.1)
HR−/HER2−	69 (23.6)	177 (13.4)	114 (10.4)	360 (13.3)
Missing data	104 (26.3)	273 (17.1)	198 (15.3)	575 (17.5)

Percentages of non-missing categories refer to the total number of patients with non-missing data

BC: breast cancer; HER2: human epidermal growth factor receptor 2; HR: hormone receptor; TNM: Tumour, node, metastasis

### Classification of breast cancer

**Fig. 2:**
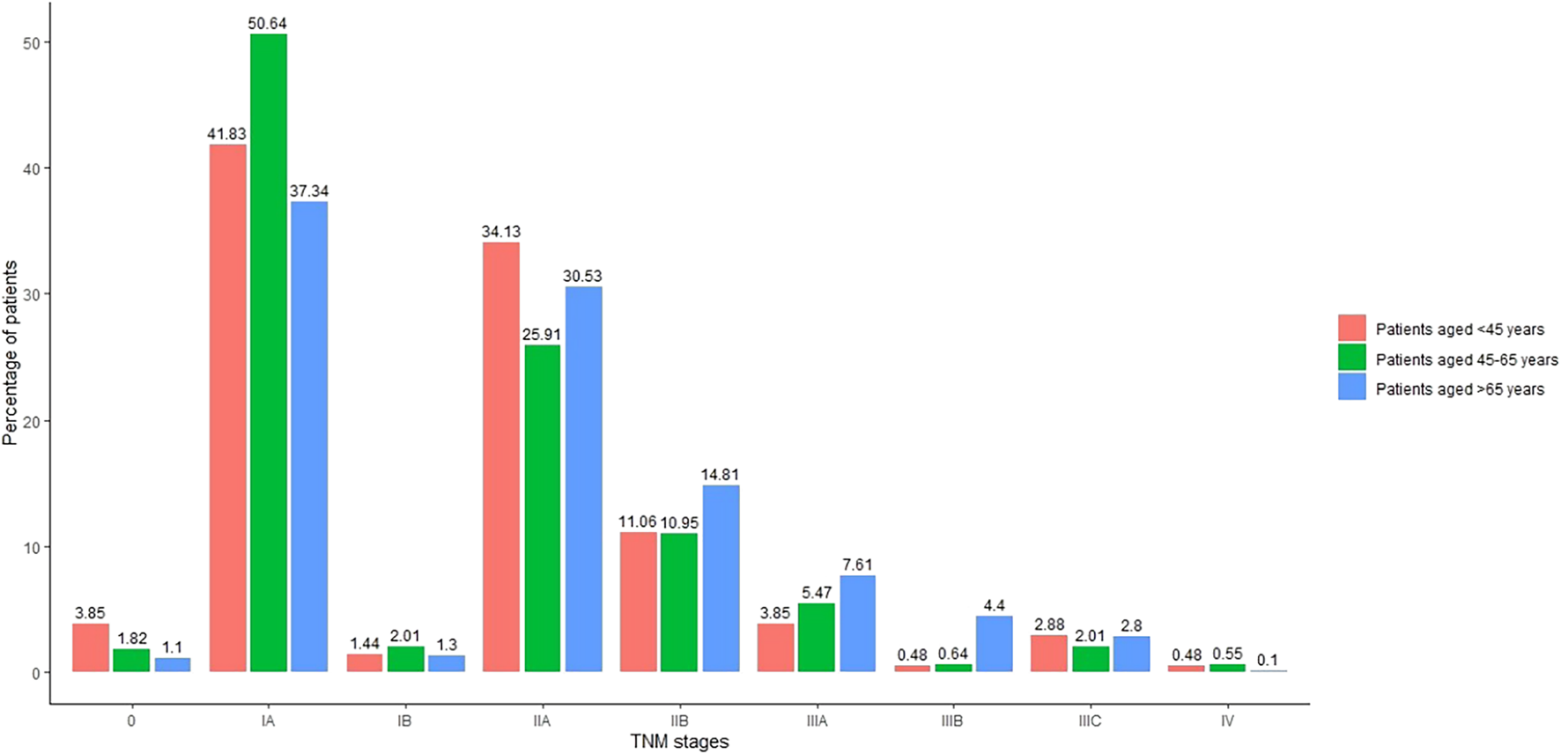
Distribution of TNM stages of breast cancer per age cohorts. TNM: Tumour, node, metastasis

**Fig. 3:**
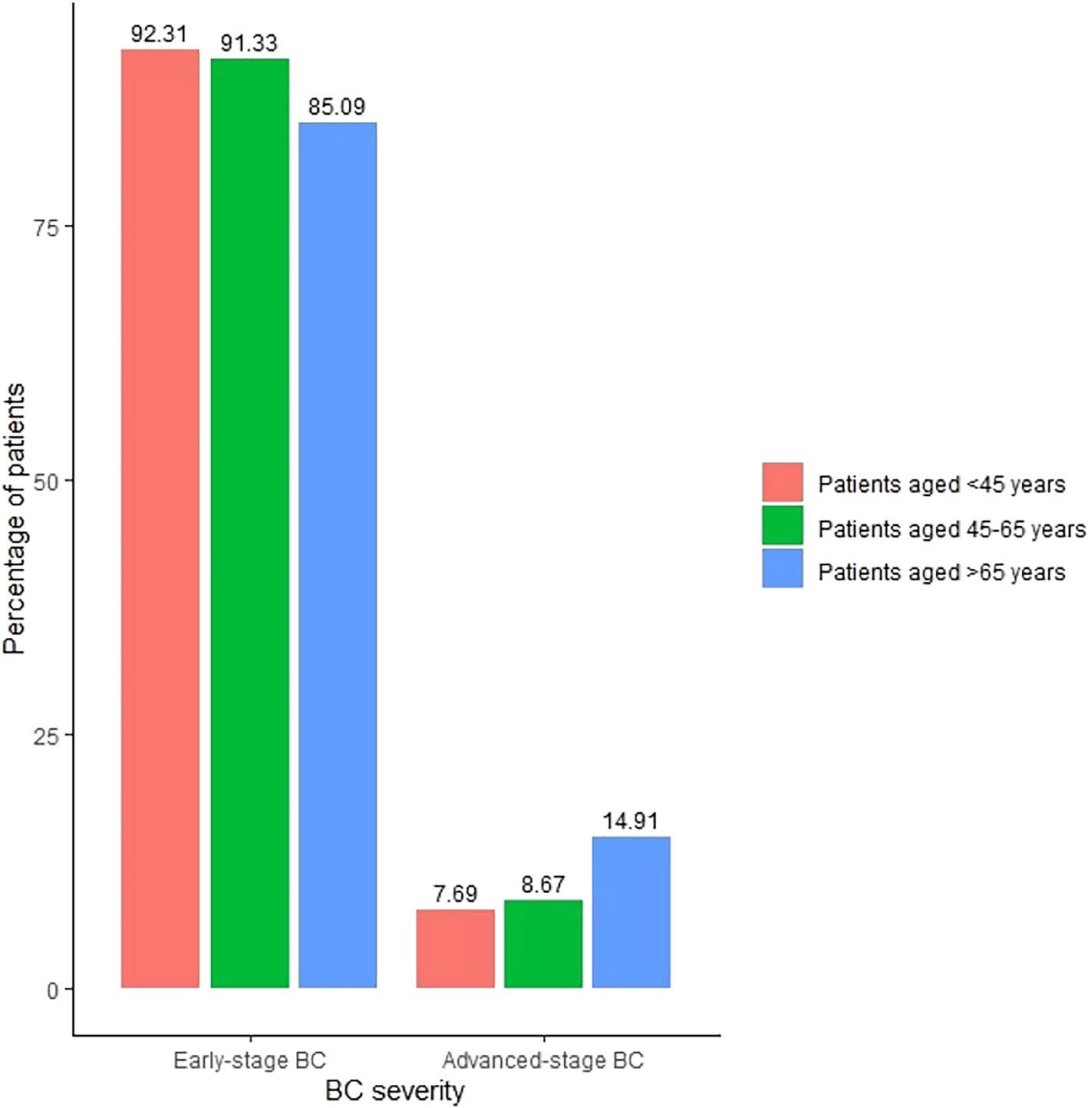
Distribution of breast cancer severity according to age cohorts. BC: breast cancer

Data on anatomic TNM stage at the time of BC diagnosis were available for 70.5% of the study population. The frequency of missing data regarding anatomic TNM stage at diagnosis was the highest in the age group < 45 years, 47.7% and the lowest in the age group > 65 years, 22.6%. The distribution of tumours by TNM classification for each age cohort is presented in Fig. 2. Among all three study cohorts, stage IA was the most prevalent TNM stage. During the study period, no significant changes were observed in the distribution of BC severity for the entire study population (P = 0.35), or within the age cohorts individually (patients aged < 45 years: P = 0.14, patients aged 45–65 years: P = 0.35, patients aged > 65 years: P = 0.49) (Supplementary Table 1). However, our analysis revealed a statistically significant difference in the distribution of BC severity across the age cohorts P < 0.001 (Fig. 3). Advanced-stage tumours were more frequent among women above age 65 years.

**Fig. 4:**
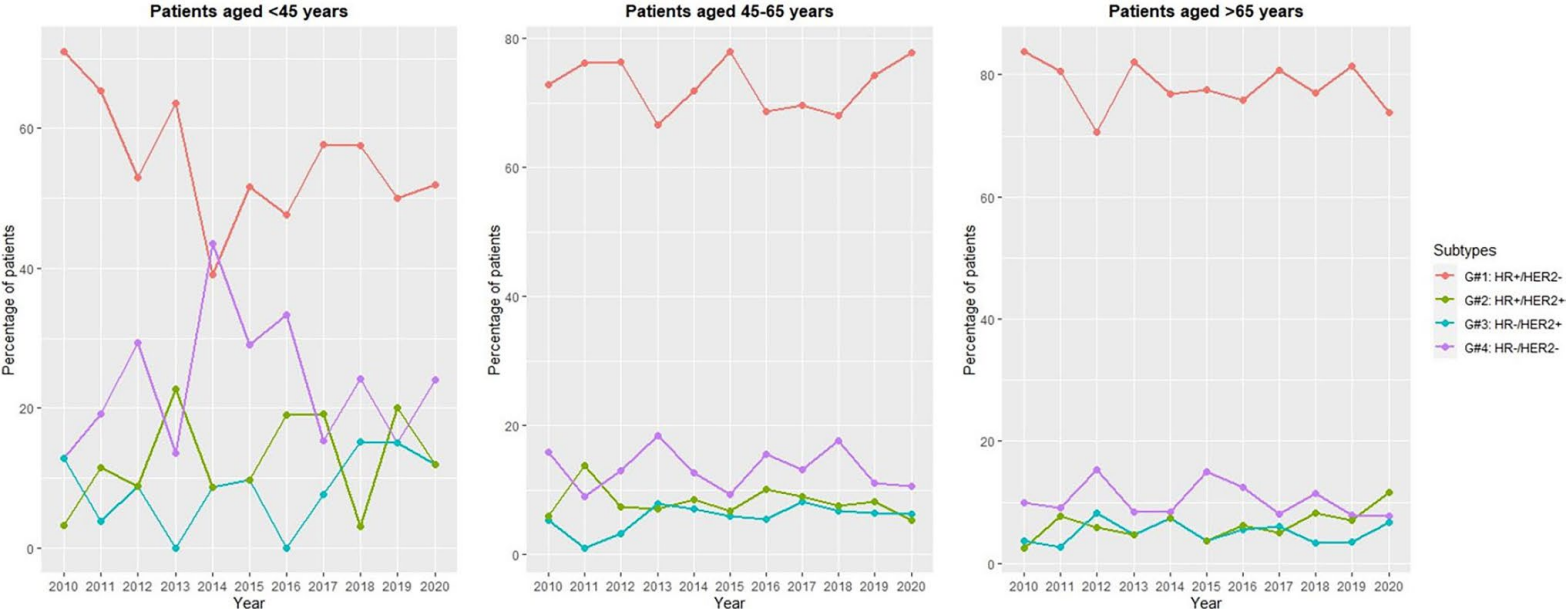
Percentage of patients according to molecular subtypes by year. HER2: human epidermal growth factor receptor 2; HR: hormone receptor

**Fig. 5:**
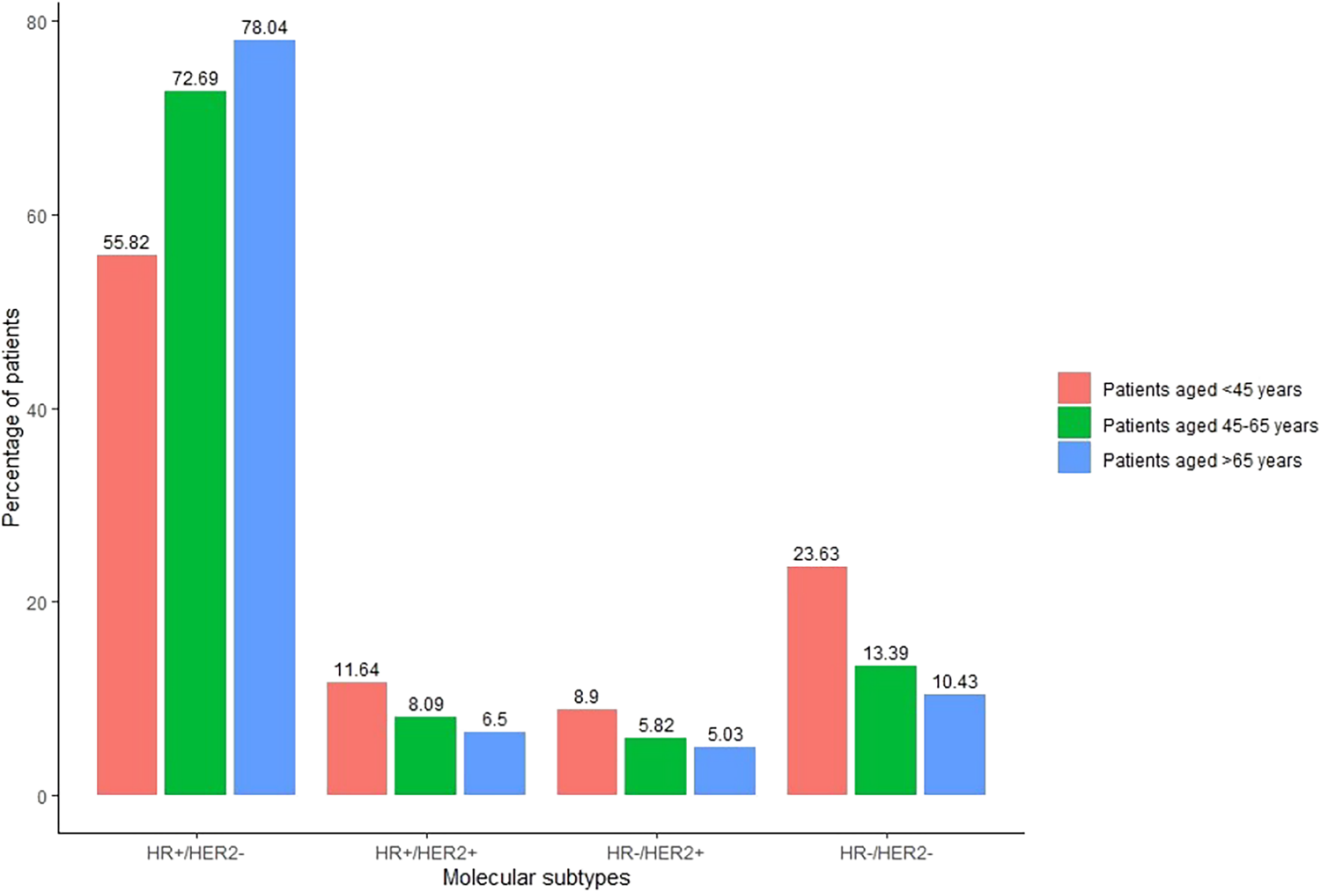
Distribution of breast cancer subtypes according to age cohorts. HER2: human epidermal growth factor receptor 2; HR: hormone receptor

Data on both HR and HER2 status at the time of BC diagnosis were available for 83.1% of the study population. The frequency of missing data regarding both HR and HER2 status at diagnosis was the highest in the age group < 45 years, 26.3% and the lowest in the age group > 65 years, 15.3%. Regarding tumour subtypes, we found that 73.0% of the newly diagnosed BC cases were HR+/HER2−, 13.3% were HR−/HER2−, 7.8% were HR+/HER2+, and 5.8% were HR−/HER2+. The annual percentage of patients according to molecular subtypes is presented in Fig. 4. Throughout the study period, there were no significant changes observed in the distribution of BC subtypes within any of the age groups (patients aged < 45 years: P = 0.39, patients aged 45–65 years: P = 0.61, patients aged > 65 years: P = 0.74) (Supplementary Table 2). However, there was a statistically significant difference in BC subtype distribution across age cohorts (P < 0.001), as shown in Fig. 5.

## Discussion

Our study brings attention to the significant proportion of BC cases occurring outside the target age group of the organised screening program. We found that only 48.6% of the tumours were detected within the screening target age range (45–65 years). In comparison, in the Netherlands, where the screening age range is 50–75 years, 62.7% of newly detected BC cases were found within the screening age group [20]. Similarly, a French study with the screening target age range of 50–74 years, found that 56.5% of the newly detected BC cases were in the screening age group [21]. These variations can be partially attributed to the differences in the age ranges of the screening populations in these countries. Furthermore, the participation rate in the screening programs have a significant impact on the number of detected tumours. During our study period, the participation rate in organised BC screening was the lowest in Hungary among these three countries ranging from 20.0% to 30.8% [22] compared to 76.8% to 79.4% in the Netherlands [23] and 49.9% to 52.1% in France [24]. Mangone et al. found that higher screening participation rates are associated with increased BC detection. They also noted that in underrepresented regions tend to have a higher frequency of advanced tumours [25]. Chen et al. suggest based on their systematic literature review and meta-analysis, that 1% increase of the attendance rate might lead to an 3% reduction in advanced stage and mortality of BC [26].

Our research revealed that within the screening population, stage I tumours accounted for the largest proportion of newly diagnosed BC cases (52.7%) Similar findings have been reported in other studies, such as 56.7% in Italy [25] and 54.3% in Norway [11]. In terms of early-stage tumours, stage II tumours were more common in relative terms among women below and above the target age range of the screening program. Advanced stage tumours were more frequent in absolute and relative terms in the older age group compared to the other age groups. This observation may be attributed to older women not being included in the organised screening program, as well as potentially lower awareness about symptoms related to BC [27], which could lead to delayed reporting of symptoms related to breast lesions.

Molecular subtype information provides crucial insights into the biological characteristics of BC [28]. In our study population, the overall distribution of molecular subtypes was comparable to findings of other population-based studies conducted in the US and Europe [16, 29]. We observed a higher proportion of HR−/HER2− and HER2+ tumours among women under 45 years of age. Timely identification of HR−/HER2− tumours is crucial due to their more aggressive clinical course and a less favourable prognosis compared to other BC subtypes [30–33]. HER2+ subtypes are also characterised by their aggressiveness and a high risk of recurrence, but targeted therapy administered in a timely manner can significantly improve patient survival [30]. The characteristics of different BC molecular subtypes, such as progression rate, radiologic appearance have a substantial impact on the likelihood of early detection. Incorporating this knowledge into screening programs could enhance early BC detection [34]. Fast-growing tumours, such as HR−/HER2− or HER2+ tumours, pose challenges in early detection due to their shorter asymptomatic time-window. Adjusting the screening frequency for individuals at higher risk of HR−/HER2− tumours (e.g., oral contraceptive users [35], premenopausal women with obesity [36]) or HER2+ (e.g., women with high mammographic density [37] or insulin resistance [38])—while carefully considering the potential for overdiagnosis and the cost-effectiveness concerns—may facilitate early tumour detection [39]. Nevertheless, further studies are required to better understand the risk factors associated with various molecular subtypes and their potential impact on the frequency of BC screening. Additionally, imaging of HR−/HER2− tumours can be difficult as they can resemble benign lesions on mammography or ultrasound [40]. It has been demonstrated that HR−/HER2− tumours are more likely to yield negative mammography results compared to other BC subtypes [41]. However, various imaging techniques, including X-ray mammography, ultrasound, digital breast tomosynthesis, magnetic resonance imaging have identified radiomic features specific to HR−/HER2− subtypes aiding their identification [42–47]. Similarly, a systematic literature review by Elias et al. identified 11 imaging features significantly associated with HER2+ overexpression of BC [48]. These imaging characteristics have the potential to enhance the BC subtype detection and even biopsy rate [44, 48]. Further studies on this phenomenon are necessary, as it holds important implications for the public health decision-making on improving BC screening programs.

The optimization of cancer screening systems necessitates continual monitoring and assessment. Real-world data, derived from health records and cancer registries, provide invaluable insights into screening program performance and disease trends. Collecting and analysing data on diverse patient and disease variables, including patient demographics and characteristics of newly diagnosed cancer cases, as reported in our research can help better understand the effectiveness of a screening program and identify gaps and areas for improvement. Monitoring of screening programs should cover the follow-up of persons with positive screening test, and patients diagnosed with breast cancer. A straightforward continuation of our research could be the survival analysis of the various BC subgroups (i.e., age groups, TNM stages and molecular subtypes) based on the data of the Hungarian National Cancer Registry.

Time-trend analysis, although unfruitful in our study possibly due to low case numbers, is essential for data-driven decisions about healthcare needs and policy development. For instance, according to the Hungarian study that observed a rise in BC incidence among females under 50 years from 2011 to 2020, it was assumed that the experienced increase is partly linked to changes in family planning practices (such as later pregnancy age and shorter breastfeeding duration), oral contraceptive usage, and other lifestyle factors like central obesity and childhood/young adult obesity, high dietary fat intake, lack of physical exercise, cigarette and alcohol consumption [14]. Raising attention to these risk factors and acknowledging their significant role during policy-making is a pivotal step towards reducing BC incidence. Additionally, understanding the shifting distribution of each molecular subtype, as evidenced by Chuaychai et al.'s research on the rising prevalence of HER2+ tumors from 2009 to 2018 [49], and in the SEER study between 2010 to 2016 [29], can also aid in alleviating the burden of BC. In the assessment of screening programs, particular attention should be given to monitoring disparities in BC characteristics across small geographical areas. Factors such as settlement size, educational level, and ethnic composition have been identified as determinants of BC incidence in Hungary [50]. A comprehensive systematic review conducted in 2022 investigated the impact of geographic accessibility and socioeconomic factors on breast cancer outcomes across various dimensions. While the results exhibit heterogeneity, the review suggests that geographic accessibility appears to more significantly affect the type of treatment received (4 out of 6 studies) than the likelihood of women undergoing screening (1 out of 4 studies). Moreover, socioeconomic factors, as measured by deprivation index, indicate that women residing in more disadvantaged areas are less likely to undergo breast-conserving surgery and more likely to undergo mastectomies compared to their counterparts in better-off areas [51]. Therefore, identifying and thoroughly examining diverse population groups could enhance the effectiveness of BC control efforts, furthermore it could offer valuable insights into associated risk factors. Our study has some limitations. The frequency of missing data on anatomic TNM stage at diagnosis varies between age groups, which may have an effect on our results. We could not categorize BC into precise molecular subtypes due to data deficiency on Ki67. Instead, HR and HER2 status were used for categorization. Although useful proxy for molecular subtyping, it may not fully capture molecular tumour characteristics. Additionally, the database could not differentiate between screening or diagnostic mammography detected cases. It is noteworthy that the final year of the observational period, 2020, coincided with the onset of the COVID-19 pandemic. Public health screening activities in Hungary were suspended between March 16 and June 1, 2020 and between April 9 and April 29, 2021. Research conducted by Elek et al. in Hungary demonstrated a 15.5% decrease in BC incidence during the pandemic [52]. This temporal overlap with the pandemic-induced disruptions in healthcare services might have exerted an influence on the outcomes observed in the present study. Despite these limitations, our study yielded invaluable data on BC cases in a large Hungarian region.

## Conclusions

In summary, our study provides valuable insights into the current state of BC screening in Hungary. By considering age, TNM stage, and molecular subtype of new BC cases, we have identified potential areas for improvement, such as extending the screening age to older cohorts and adjusting the screening frequency based on the risk for HR−/HER2− or HER2+ tumours. The continuous scrutiny and evaluation of these screening programs using real-world data is fundamental to assess intervention impacts and refine screening systems.

### Supplementary Information

Below is the link to the electronic supplementary material.

Supplementary file1 (DOCX 21 KB)

Supplementary file2 (DOCX 19 KB)

## Data Availability

The dataset analysed in this study is available from the corresponding author upon reasonable request.

## References

[1] Sung H, Ferlay J, Siegel RL, Laversanne M, Soerjomataram I, Jemal A, Bray F (2021). Global cancer statistics 2020: GLOBOCAN estimates of incidence and mortality worldwide for 36 cancers in 185 countries. CA Cancer J Clin.

[2] World Health Organization. Regional Office for Europe. Screening programmes: a short guide. Increase effectiveness, maximize benefits and minimize harm. Copenhagen: World Health Organization. 2020. Regional Office for Europe. https://apps.who.int/iris/handle/10665/330829. Accessed 20 April 2023

[3] European Commission Initiative on Breast Cancer (ECIBC). European guidelines on breast cancer screening and diagnosis. 2019. https://healthcare-quality.jrc.ec.europa.eu/ecibc/european-breast-cancer-guidelines. Accessed 20 April 2023

[4] Forrai G, Kovács E, Ambrózay É, Barta M, Borbély K, Lengyel Z, Ormándi K, Péntek Z, Tasnádi T, Sebő É (2020). Use of imaging methods in the current screening, diagnostics and treatment of breast cancer – 4th Breast Cancer Consensus Conference. Magy Onkol.

[5] Dafni U, Tsourti Z, Alatsathianos I (2019). Breast cancer statistics in the European Union: incidence and survival across European countries. Breast Care.

[6] Colditz GA, Rosner BA, Chen WY, Holmes MD, Hankinson SE (2004). Risk factors for breast cancer according to estrogen and progesterone receptor status. J Natl Cancer Inst.

[7] Lofterød T, Frydenberg H, Flote V, Eggen AE, McTiernan A, Mortensen ES, Akslen LA, Reitan JB, Wilsgaard T, Thune I (2020). Exploring the effects of lifestyle on breast cancer risk, age at diagnosis, and survival: the EBBA-Life study. Breast Cancer Res Treat.

[8] Kamińska M, Ciszewski T, Łopacka-Szatan K, Miotła P, Starosławska E (2015). Breast cancer risk factors. Prz Menopauzalny.

[9] Chen HL, Zhou MQ, Tian W, Meng KX, He HF (2016). Effect of age on breast cancer patient prognoses: a population-based study using the SEER 18 database. PLoS ONE.

[10] Pelofi G, Martin X, Barben J, Jouanny P (2022). Interest of individual breast cancer screening by mammography in women aged over 75 years. Geriatr Psychol Neuropsychiatr Vieil.

[11] Johansson ALV, Trewin CB, Hjerkind KV, Ellingjord-Dale M, Johannesen TB, Ursin G (2019). Breast cancer-specific survival by clinical subtype after 7 years follow-up of young and elderly women in a nationwide cohort. Int J Cancer.

[12] Kataoka A, Iwamoto T, Tokunaga E, Tomotaki A, Kumamaru H, Miyata H, Niikura N, Kawai M, Anan K, Hayashi N, Masuda S, Tsugawa K, Aogi K, Ishida T, Masuoka H, Iijima K, Kinoshita T, Nakamura S, Tokuda Y (2016). Young adult breast cancer patients have a poor prognosis independent of prognostic clinicopathological factors: a study from the Japanese Breast Cancer Registry. Breast Cancer Res Treat.

[13] Kim HJ, Kim S, Freedman RA, Partridge AH (2022). The impact of young age at diagnosis (age <40 years) on prognosis varies by breast cancer subtype: a U.S. SEER database analysis. Breast.

[14] Kiss Z, Kocsis J, Nikolényi A, Horváth Z, Knollmajer K, Benedek A, Várnai M, Polányi Z, Kovács KA, Berta A, Köveskuti I, Karamousouli E, Szabó TG, Rokszin G, Fábián I, Bartókné Tamás R, Surján O, Fürtős D, Surján G, Kenessey I, Weber A, Barcza Z, Berki T, Vokó Z, Dózsa C, Dank M, Boér K (2023). Opposite trends in incidence of breast cancer in young and old female cohorts in Hungary and the impact of the Covid-19 pandemic: a nationwide study between 2011–2020. Front Oncol.

[15] Cai S, Zuo W, Lu X, Gou Z, Zhou Y, Liu P, Pan Y, Chen S (2020). The prognostic impact of age at diagnosis upon breast cancer of different immunohistochemical subtypes: a surveillance, epidemiology, and end results (SEER) population-based analysis. Front Oncol.

[16] Cortet M, Bertaut A, Molinié F, Bara S, Beltjens F, Coutant C, Arveux P (2018). Trends in molecular subtypes of breast cancer: description of incidence rates between 2007 and 2012 from three French registries. BMC Cancer.

[17] Kramp LJ, Mathiak M, Behrens HM, Schäfer FW, van Mackelenbergh M, Röcken C (2022). The age-specific differences in histopathological tumor characteristics and TNM classification of breast carcinomas in Quality assured mamma diagnostic (QuaMaDi) program in the state of Schleswig-Holstein in Germany. J Cancer Res Clin Oncol.

[18] Tzikas AK, Nemes S, Linderholm BK (2020). A comparison between young and old patients with triple-negative breast cancer: biology, survival and metastatic patterns. Breast Cancer Res Treat.

[19] Giuliano AE, Edge SB, Hortobagyi GN (2018). Eighth Edition of the AJCC cancer staging manual: breast cancer. Ann Surg Oncol.

[20] van der Meer DJ, Kramer I, van Maaren MC, van Diest PJ, Linn CS, Maduro JH, Strobbe LJA, Siesling S, Schmidt MK, Voogd AC (2021). Comprehensive trends in incidence, treatment, survival and mortality of first primary invasive breast cancer stratified by age, stage and receptor subtype in the Netherlands between 1989 and 2017. Int J Cancer.

[21] Hassaine Y, Jacquet E, Seigneurin A, Delafosse P (2022). Evolution of breast cancer incidence in young women in a French registry from 1990 to 2018: towards a change in screening strategy?. Breast Cancer Res.

[22] Laczó A, Bódis J, Bogner P, Molnár K, Vajda R, Pónusz-Kovács D, Elmer D, Kajos FL, Csákvári T, Kívés Z, Boncz I (2022). Participation indicators of organized mammography screening in Hungary between 2012–2021. Magy Onkol.

[23] Gong J, Kampadellis G, Kong Q, Spijker W (2023). Factors determining non-attendance in breast cancer screening among women in the Netherlands: a national study. Health Promot Int.

[24] Statista Research Department. Evolution of the rate of the participation in the organized breast screening program in France from 2014 to 2017. https://www.statista.com/statistics/964518/breast-cancer-screening-national-participation-rate-france/ Accessed 20 Apr 2023

[25] Mangone L, Bisceglia I, Michiara M, Musolino A, Mazzoleni G, Caldarella A, Minerba S, Cascone G, Bella F, Dinaro Y, Pau L, Pinto C (2022). Breast cancer in Italy: stage and region distribution. Breast Cancer.

[26] Chen TH, Yen AM, Fann JC, Gordon P, Chen SL, Chiu SY, Hsu CY, Chang KJ, Lee WC, Yeoh KG, Saito H, Promthet S, Hamashima C, Maidin A, Robinson F, Zhao LZ (2017). Clarifying the debate on population-based screening for breast cancer with mammography: a systematic review of randomized controlled trials on mammography with Bayesian meta-analysis and causal model. Medicine.

[27] Linsell L, Forbes LJ, Kapari M, Burgess C, Omar L, Tucker L, Ramirez AJ (2009). A randomised controlled trial of an intervention to promote early presentation of breast cancer in older women: effect on breast cancer awareness. Br J Cancer.

[28] Prat A, Pineda E, Adamo B, Galván P, Fernández A, Gaba L, Díez M, Viladot M, Arance A, Muñoz M (2015). Clinical implications of the intrinsic molecular subtypes of breast cancer. Breast.

[29] Acheampong T, Kehm RD, Terry MB, Argov EL, Tehranifar P (2020). Incidence trends of breast cancer molecular subtypes by age and race/ethnicity in the US from 2010 to 2016. JAMA Netw Open.

[30] Loibl S, Gianni L (2017). HER2-positive breast cancer. Lancet.

[31] Arpino G, Milano M, De Placido S (2015). Features of aggressive breast cancer. Breast.

[32] Manjunath M, Choudhary B (2021). Triple-negative breast cancer: a run-through of features, classification and current therapies. Oncol Lett.

[33] Medina MA, Oza G, Sharma A, Arriaga LG, Hernández Hernández JM, Rotello VM, Ramirez JT (2020). Triple-negative breast cancer: a review of conventional and advanced therapeutic strategies. Int J Environ Res Public Health.

[34] Ding L, Greuter MJW, Truyen I, Goossens M, Van der Vegt B, De Schutter H, Van Hal G, de Bock GH (2022). Effectiveness of organized mammography screening for different breast cancer molecular subtypes. Cancers.

[35] Barańska A, Dolar-Szczasny J, Kanadys W, Kinik W, Ceglarska D, Religioni U, Rejdak R (2022). Oral contraceptive use and breast cancer risk according to molecular subtypes status: a systematic review and meta-analysis of case-control studies. Cancers.

[36] Torres-de la Roche LA, Steljes I, Janni W, Friedl TWP, De Wilde RL (2020). The association between obesity and premenopausal breast cancer according to intrinsic subtypes - a systematic review. Geburtshilfe Frauenheilkd.

[37] Bai S, Song D, Chen M, Lai X, Xu J, Dong F (2023). The association between mammographic density and breast cancer molecular subtypes: a systematic review and meta-analysis. Clin Radiol.

[38] Capasso I, Esposito E, de Laurentiis M, Maurea N, Cavalcanti E, Botti G, Petrillo A, Montella M, D'Aiuto M, Coppola C, Crispo A, Grimaldi M, Frasci G, Fucito A, Ciliberto G, D'Aiuto G (2014). Metabolic syndrome-breast cancer link varies by intrinsic molecular subtype. Diabetol Metab Syndr.

[39] Perron L, Chang SL, Daigle JM, Vandal N, Theberge I, Diorio C, Lemieux J, Pelletier E, Brisson J (2019). Breast cancer subtype and screening sensitivity in the Quebec Mammography Screening Program. J Med Screen.

[40] Azzam H, Kamal R, El-Assaly H, Omer L (2020). The value of dynamic contrast-enhanced MRI in the diagnosis and management of triple-negative breast cancer. Egypt J Radiol Nucl Med.

[41] Lohitvisate W, Pummee N, Kwankua A (2023). Mammographic and ultrasonographic features of triple-negative breast cancer compared with non-triple-negative breast cancer. J Ultrasound.

[42] Rashmi S, Kamala S, Murthy SS, Kotha S, Rao YS, Chaudhary KV (2018). Predicting the molecular subtype of breast cancer based on mammography and ultrasound findings. Indian J Radiol Imaging.

[43] Huang J, Lin Q, Cui C, Fei J, Su X, Li L, Ma J, Zhang M (2020). Correlation between imaging features and molecular subtypes of breast cancer in young women (≤30 years old). Jpn J Radiol.

[44] Ian TWM, Tan EY, Chotai N (2021). Role of mammogram and ultrasound imaging in predicting breast cancer subtypes in screening and symptomatic patients. World J Clin Oncol.

[45] Chen IE, Lee-Felker S (2023). Triple-negative breast cancer: multimodality appearance. Curr Radiol Rep.

[46] Sha YS, Chen JF (2022). MRI-based radiomics for the diagnosis of triple-negative breast cancer: a meta-analysis. Clin Radiol.

[47] Lee SH, Chang JM, Shin SU, Chu AJ, Yi A, Cho N, Moon WK (2017). Imaging features of breast cancers on digital breast tomosynthesis according to molecular subtype: association with breast cancer detection. Br J Radiol.

[48] Elias SG, Adams A, Wisner DJ, Esserman LJ, van’t Veer LJ, Mali WP, Gilhuijs KG, Hylton NM (2014). Imaging features of HER2 overexpression in breast cancer: a systematic review and meta-analysis. Cancer Epidemiol Biomarkers Prev.

[49] Chuaychai A, Sriplung H (2022). A rapid rise in hormone receptor-positive and HER2-positive breast cancer subtypes in Southern Thai women: a population-based study in Songkhla. PLoS ONE.

[50] Sándor J, Havasi V, Kiss I, Szücs M, Brázay L, Sebestyén A, Ember I (2002). Small area inequalities in breast cancer mortality and screening. Magy Onkol.

[51] Conti B, Bochaton A, Charreire H, Kitzis-Bonsang H, Desprès C, Baffert S, Ngô C (2022). Influence of geographic access and socioeconomic characteristics on breast cancer outcomes: a systematic review. PLoS ONE.

[52] Elek P, Csanádi M, Fadgyas-Freyler P, Gervai N, Oross-Bécsi R, Szécsényi-Nagy B, Tatár M, Váradi B, Zemplényi A (2022). Heterogeneous impact of the COVID-19 pandemic on lung, colorectal and breast cancer incidence in Hungary: results from time series and panel data models. BMJ Open.

