# Supplementary material for: Breast cancer stage and molecular subtype distribution: real-world insights from a regional oncological center in Hungary
Source: Discov Oncol. 2024 Jun 22;15:240. doi: 10.1007/s12672-024-01096-9 (PMC11193705; doi:10.1007/s12672-024-01096-9)
Supplement: Supplementary file 1 — Supplementary file1 (DOCX 21 KB) [file 12672_2024_1096_MOESM1_ESM.docx]

**Supplementary Table 1. Annual number of newly diagnosed breast cancer cases during the study period per age and anatomic TNM stages**

| **Year**  Age cohorts | **Anatomic TNM stages**  (N of subjects) | | | | | | | | | **BC severity**  (N of subjects) | |
| --- | --- | --- | --- | --- | --- | --- | --- | --- | --- | --- | --- |
|  | **0** | **IA** | **IB** | **IIA** | **IIB** | **IIIA** | **IIIB** | **IIIC** | **IV** | **Early-stage** | **Advanced-stage** |
| **2010**  Patients aged <45 y  Patients aged 45-65 y  Patients aged >65 y  Total | 2  4  1  7 | 12  54  26  92 | 0  4  0  4 | 9  32  23  64 | 1  16  11  28 | 1  4  6  11 | 0  0  3  3 | 0  2  3  5 | 0  0  0  0 | 24  110  61  195 | 1  6  12  19 |
| **2011**  Patients aged <45 y  Patients aged 45-65 y  Patients aged >65 y Total | 1  2  0  3 | 5  42  22  69 | 0  1  1  2 | 5  25  19  49 | 4  12  11  27 | 0  4  8  12 | 1  0  2  3 | 1  3  3  7 | 1  0  0  1 | 15  82  53  150 | 3  7  13  23 |
| **2012**  Patients aged <45 y  Patients aged 45-65 y  Patients aged >65 y Total | 2  0  2  4 | 13  55  26  94 | 0  0  0  0 | 10  28  27  65 | 3  8  14  25 | 1  11  9  21 | 0  2  4  6 | 0  3  5  8 | 0  1  0  1 | 28  91  69  188 | 1  17  18  36 |
| **2013**  Patients aged <45 y  Patients aged 45-65 y  Patients aged >65 y Total | 0  2  2  4 | 7  54  31  92 | 0  1  0  1 | 5  22  21  48 | 1  5  13  19 | 0  6  5  11 | 0  0  2  2 | 1  3  2  6 | 0  0  0  0 | 13  84  67  164 | 1  9  9  19 |
| **2014**  Patients aged <45 y  Patients aged 45-65 y  Patients aged >65 y Total | 0  5  2  7 | 12  74  29  115 | 0  4  0  4 | 6  26  40  72 | 0  8  5  13 | 0  9  3  12 | 0  0  4  4 | 0  2  0  2 | 0  0  0  0 | 18  117  76  211 | 0  11  7  18 |
| **2015**  Patients aged <45 y  Patients aged 45-65 y  Patients aged >65 y Total | 0  2  0  2 | 7  57  41  105 | 0  2  0  2 | 6  20  32  58 | 3  14  16  33 | 4  5  5  14 | 0  1  5  6 | 1  0  1  2 | 0  1  0  1 | 16  95  89  200 | 5  7  11  23 |
| **2016**  Patients aged <45 y  Patients aged 45-65 y  Patients aged >65 y Total | 1  2  1  4 | 3  50  38  91 | 0  2  2  4 | 6  30  37  73 | 2  14  22  38 | 0  3  6  9 | 0  0  5  5 | 1  3  4  8 | 0  3  0  3 | 12  98  100  210 | 1  9  15  25 |
| **2017**  Patients aged <45 y  Patients aged 45-65 y  Patients aged >65 y Total | 0  0  2  2 | 6  43  36  85 | 0  2  4  6 | 4  29  21  54 | 4  9  14  27 | 0  5  8  13 | 0  2  2  4 | 0  2  4  6 | 0  0  0  0 | 14  83  77  174 | 0  9  14  23 |
| **2018**  Patients aged <45 y  Patients aged 45-65 y  Patients aged >65 y Total | 0  1  0  1 | 8  46  48  102 | 1  2  1  4 | 9  29  32  70 | 1  8  17  26 | 1  5  10  16 | 0  2  6  8 | 1  1  1  3 | 0  1  0  1 | 19  86  98  203 | 2  9  17  28 |
| **2019**  Patients aged <45 y  Patients aged 45-65 y  Patients aged >65 y Total | 0  0  1  1 | 6  37  37  80 | 1  1  2  4 | 5  22  23  50 | 1  14  17  32 | 0  5  9  14 | 0  0  6  6 | 0  2  3  5 | 0  0  0  0 | 13  74  80  167 | 0  7  18  25 |
| **2020**  Patients aged <45 y  Patients aged 45-65 y  Patients aged >65 y Total | 2  2  0  4 | 8  43  39  90 | 1  3  3  7 | 6  21  30  57 | 3  12  8  23 | 1  3  7  11 | 0  0  5  5 | 1  1  2  4 | 0  0  1  1 | 20  81  80  181 | 2  4  15  21 |

BC: breast cancer; TNM: Tumour, node, metastasis; y: year
