# Supplementary material for: Breast cancer stage and molecular subtype distribution: real-world insights from a regional oncological center in Hungary
Source: Discov Oncol. 2024 Jun 22;15:240. doi: 10.1007/s12672-024-01096-9 (PMC11193705; doi:10.1007/s12672-024-01096-9)
Supplement: Supplementary file 2 — Supplementary file2 (DOCX 19 KB) [file 12672_2024_1096_MOESM2_ESM.docx]

**Supplementary Table 2.** **Annual number of newly diagnosed breast cancer cases during the study period per age and molecular subtype**

| **Year**  Age cohorts | **Molecular subtypes** (N of subjects) | | | |
| --- | --- | --- | --- | --- |
|  | **HR+/HER2-** | **HR+/HER2+** | **HR-/HER2+** | **HR-/HER2-** |
| **2010**  Patients aged <45 y  Patients aged 45-65 y  Patients aged >65 y Total | 22  97  67  186 | 1  8  2  11 | 4  7  3  14 | 4  21  8  33 |
| **2011**  Patients aged <45 y  Patients aged 45-65 y  Patients aged >65 y Total | 17  77  62  156 | 3  14  6  23 | 1  1  2  4 | 5  9  7  21 |
| **2012**  Patients aged <45 y  Patients aged 45-65 y  Patients aged >65 y Total | 18  94  60  172 | 3  9  5  17 | 3  4  7  14 | 10  16  13  39 |
| **2013**  Patients aged <45 y  Patients aged 45-65 y  Patients aged >65 y Total | 14  76  69  159 | 5  8  4  17 | 0  9  4  13 | 3  21  7  31 |
| **2014**  Patients aged <45 y  Patients aged 45-65 y  Patients aged >65 y Total | 9  102  73  184 | 2  12  7  21 | 2  10  7  19 | 10  18  8  36 |
| **2015**  Patients aged <45 y  Patients aged 45-65 y  Patients aged >65 y Total | 16  92  83  191 | 3  8  4  15 | 3  7  4  14 | 9  11  16  36 |
| **2016**  Patients aged <45 y  Patients aged 45-65 y  Patients aged >65 y Total | 10  88  97  195 | 4  13  8  25 | 0  7  7  14 | 7  20  16  43 |
| **2017**  Patients aged <45 y  Patients aged 45-65 y  Patients aged >65 y Total | 15  85  80  180 | 5  11  5  21 | 2  10  6  18 | 4  16  8  28 |
| **2018**  Patients aged <45 y  Patients aged 45-65 y  Patients aged >65 y Total | 19  81  94  194 | 1  9  10  20 | 5  8  4  17 | 8  21  14  43 |
| **2019**  Patients aged <45 y  Patients aged 45-65 y  Patients aged >65 y Total | 10  81  92  183 | 4  9  8  21 | 3  7  4  14 | 3  12  9  24 |
| **2020**  Patients aged <45 y  Patients aged 45-65 y  Patients aged >65 y Total | 13  88  76  177 | 3  6  12  21 | 3  7  7  17 | 6  12  8  26 |

HER2: human epidermal growth factor receptor 2; HR: hormone receptor; y: year
